# Home is best: Why women in rural Zimbabwe deliver in the community

**DOI:** 10.1371/journal.pone.0181771

**Published:** 2017-08-09

**Authors:** Munyaradzi Kenneth Dodzo, Marvellous Mhloyi

**Affiliations:** Centre for Population Studies, University of Zimbabwe, Harare, Zimbabwe; Jhpiego, UNITED STATES

## Abstract

Maternal mortality in Zimbabwe has unprecedentedly risen over the last two and half decades although a decline has been noted recently. Many reasons have been advanced for the rising trend, including deliveries without skilled care, in places without appropriate or adequate facilities to handle complications. The recent decline has been attributed to health systems strengthening through a multi-donor pooled funding mechanism. On the other hand, the proportion of community deliveries has also been growing steadily over the years and in this study we investigate why. We used twelve (12) focus group discussions with child-bearing women and eight (8) key informant interviews (KIIs). Four (4) were traditional birth attendants and four (4) were spiritual birth attendants. A thematic approach was used to analyse the data in Ethnography software. The study shows that women prefer community deliveries due to perceived low economic, social and opportunity costs involved; pliant and flexible services offered; and diminishing quality and appeal of institutional maternity services. We conclude that rural women are very economic, logical and rational in making choices on place of delivery. Delivering in the community offers financial, social and opportunity advantages to disenfranchised women, particularly in remote rural areas. We recommend for increased awareness of the dangers of community deliveries; establishment of basic obstetric care facilities in the community and more efficient emergency referral systems. In the long-term, there should be a sustainable improvement of the public health delivery system to make it accessible, affordable and usable by the public.

## Introduction and study context

When pregnancy and child birth are planned and intended they are usually associated with joy and celebration, at least in a Zimbabwean context. A newly born child is regarded as a gift to the family and the world at large. In most cases, pregnant women expect the successful delivery of a healthy baby. However, maternal mortality remains pervasive in rural settings. In many cases, pregnancies end in fatalities, killing either the mother, baby or both. The causes are numerous, the instances diverse and the circumstances complex. One known cause of high maternal mortality is community delivery without skilled staff, equipment and drugs, in conditions that are not conducive for safe delivery [1,2,3]. Little is known about the rationale of community deliveries apart from the negative issues associated with health facilities, which include cost, poor attitudes of hospital staff, waiting times and long distance. This paper aims to explore some of the reasons why community deliveries are getting more attractive and being preferred by women.

Zimbabwe is a land locked country in Southern Africa, which has 10 administrative provinces, 8 of which are rural. Most Zimbabweans, 67%, live in rural areas [4] where availability, accessibility and affordability are key issues impinging on health utilization. The country inherited the Rhodesian health care system at independence in 1980 which had racially segregated health services. The distribution of resources was highly skewed towards hospital service provision for the small white population at the expense of the indigenous population. Post-independent Zimbabwe saw government trying to redress the existing inequalities by investing especially in rural health services because health was considered an integral part of development and a human right. Although there are currently over 1,500 health facilities around the country, there are still hard to reach areas that are not yet serviced [5]. Zimbabwe’s health system is dominated by the public sector, which provides an estimated 65% of health care services in the country. The mission (faith-based) sector plays a major role in rural areas courtesy of the colonial legacy while the private-for-profit sector is predominantly in urban areas. Some facilities are operated by municipalities and receive block grants from government. Most communities live within a 5km radius from their nearest health facilities, whilst 23% live between 5 and 10km and 17% are over 10km from their nearest health centre [5]. Health care in Zimbabwe has been categorized as pluralistic due to the existence of both traditional and biomedical systems [6]. Historically, Zimbabwe has focused on Primary Health Care, with a strong emphasis on community-based approaches, complemented by robust referral systems and facilities [5].

Zimbabwe’s maternal mortality ratio (MMR), which measures the number of maternal deaths per 100,000 live births, steadily rose from 283 in 1994 [7] to 694 in 1999 [8]. In 2006 it was pegged at 578 [9], then 725 in 2009 [10]. In 2010, it was estimated at 960 [11] while the census of 2012, pegged it at 1,024 [4]. In 2014, it was pegged at 614 [12] and currently stands at 650 [13], indicating a reversal of the falling trend. Although the data sources use different methodologies, the fact remains that there has been a general increase in MMR over the years. Recent statistics, however, may indicate the beginning of another era of rising maternal mortality after a noticeable decline. Undeniably, the maternal mortality ratio for Zimbabwe is intolerably high. Furthermore, the oscillating trends suggest a lack of sustainable interventions to keep it on a decline but these patterns appear to reflect the level of development and the socio-economic performance of the country, as observed by the World Bank and others [14,15].

At the turn of the century, Zimbabwe went through a period of political plurality and tensions in governance systems. The sequel was social upheaval and economic fragility, characterized by poor performance of key social sectors, including health. On the economic front, households and individuals were not spared as agriculture failed, industry collapsed, unemployment increased, aggregate expenditure declined, government’s capacity to collect revenue shrank while fiscal and monetary policies failed. The height of this socio-economic crisis came in 2008 when inflation rates reached 231 million percent, the local currency failed, while trade and economic activity stagnated [16]. As such, government capacity to provide services was crippled and the allure of health facilities waned. In 2009, socio-economic stability was restored when the government introduced a multi-currency regime dominated by the United States dollar [16]. Nevertheless, some of the problems prevailed because some macro-economic and governance fundamentals were not addressed or are still being contested.

In the backdrop of such socio-economic upheaval, reproduction did not stop, spelling negative consequences for key maternal health indicators, including MMR. Around 2010, the major donors in the country created a multi-million dollar pooled funding mechanism to stimulate the social sectors, including health, education, protection and water, sanitation and hygiene. Among these pooled funding mechanisms was the Health Transition Fund (HTF) [17]. It sought to improve maternal and new-born health by procuring and distributing drugs, equipment, sundries and paying top-up salaries for health workers. Although it mainly catered to supply-side issues, it did nothing to create demand, change attitudes and improve motivation to seek facility-based health services. It is generally believed that this intervention led to the reduction in the maternal mortality ratio from 750 in 2010 [10] to 614 deaths per 100,000 live births in 2014 [12].

Assuming that the HTF was responsible for the huge decline in MMR recorded within 4 years, it can also be postulated that much more could have been achieved had it also addressed the community determinants of maternal mortality. Among these possible interventions, one would be increasing the proportion of deliveries occurring in health facilities. Most studies on maternal mortality seek to establish the levels and causes but the social determinants, including factors of choice of place of delivery, have not been fully investigated in context. It is generally agreed that maternal mortality is not a straightforward phenomenon in low-income economies as many factors come into play. Although recent trends of declining MMR in Zimbabwe have been attributed to huge improvements made by the HTF in health infrastructure, human resources, drugs and equipment, it is not clear whether the relationship is causal or sustainable. Already, recent results show that MMR is higher than in 2014 [12,13]. A grey area remains that of the demand side factors, including community and individual beliefs, attitudes and practices.

Place of delivery is usually a huge determinant of maternal outcome [1]. Yet the evidence shows that community deliveries in Zimbabwe continued to rise steadfastly from 23% in 1999 to 31% in 2006 [8,9] and 35% in 2011 [11]. It can be noted that, even when the MMR finally started to decline for the first time in three decades to 614 deaths per 100,000 live births [12], community deliveries remained on the ascendancy [11]. This is in spite of the fact that community birth attendants, who include traditional and spiritual birth attendants, are outlawed. A response lag can be assumed in light of recent results, which show that community deliveries finally declined to 28% [13]. Literature shows that place of delivery is a process indicator that affects not only maternal but also perinatal outcomes [1]. Women who deliver in facilities have better outcomes than those delivering in the community, assuming health facilities have the trained staff, infrastructure and standardized delivery protocols needed to manage obstetric complications.

While analysing retrospective maternal mortality data for the period 1999–2008, Dodzo [18] established that 25% of deceased women had delivered in the community. In a bivariate analysis of survey data for 2008, he found that 31% of the women had delivered in the community. These levels are similar to those reported by consecutive rounds of the Zimbabwe Demographic and Health Survey (ZDHS) [8,9,11]. He also found that place of delivery was strongly associated with maternal outcomes, p<0.001, since over 50% of the casualties, compared to only 31% among survivors, had delivered in the community [18]. This shows the high risk associated with community deliveries. Nevertheless, institutional delivery can increase the risk of maternal mortality if there is poor quality of services, calibre of providers and capacity of facilities. In Malawi, women delivering in facilities demonstrated a significantly higher risk of experiencing a maternal death than those delivering in the community [19]. Among several possible reasons, this was due to high risk selection [20] coupled with lack of institutional capacity to offer quality care to high-risk women [2]. In West Africa, women who received sub-standard institutional care were more likely to die than those receiving optimal care [21]. Further, hospital deficiencies such as high vacancy rates and staff absenteeism are risk factors [22].

Notwithstanding shaky evidence, the possibility that mothers might be harmed at facilities due to poor infection control or other human errors cannot be ruled out [23]. Push factors that keep women away from health facilities are usually cost, poor attitudes of hospital staff, long waiting time, long distance and bad experiences during their last hospital deliveries [3]. While this is excellent evidence on the push factors of health facilities, what attracts women to deliver in the community remains largely unknown. Apparently, the loss of allure by the public health system has been fronted as the only reason for community deliveries. It helps to understand the increasing pull of the community as a place of delivery.

In Zimbabwe, the ZDHS is the perhaps the only national survey that attempts to look at factors of health care utilization. It indicates that problems in accessing health care include lack of money to pay for treatment (50%), distance to health facility (34%), permission to seek health care (8%) and the desire for company (14%) [11]. Financial problems were cited by women who had five or more living children (68%), were divorced, separated or widowed (64%), resided in rural areas (59%), lived in Matabeleland South (65%), had no education (75%) or were in the lowest wealth quintile (70%). Concerns about distance to health facility were cited more by women residing in rural areas (49%) than in urban areas (11%). These findings demonstrate that women in rural areas face multiple challenges that can lead to community deliveries. Further, the ZDHS [11] shows that 56%, 3% and 7% of deliveries occurred in public, private and mission health facilities respectively. Women aged 20–34 years are likely to deliver in a health facility (66%) compared to younger (64%) and older women (61%). Sixty percent of higher order births (6+) occurred in the community compared to only 25% of first order births. Antenatal care attendance is positively related to institutional delivery. Only 28% of women with no antenatal care, compared to 74% with four or more visits delivered in a health facility.

Place of residence is another factor of place of delivery [11]. The analysis showed that in urban areas 85% of births occurred in a health facility compared to only 57% in rural areas. As a characteristic feature, DHS reports do not go further to establish the reasons nor test for significance of differences. Education emerged as another correlate of place of delivery, whereby 95% of births to mothers with at least secondary education occurred in health facilities compared to only 36% among those with no education [5]. Household wealth appears to influence choice of place of delivery [5]. Mothers in the highest wealth quintile were twice as likely (90%) to give birth in a health facility as those in the lowest wealth quintile (46%). This statistical distribution of place of delivery does not reveal why rural women prefer to deliver in the community.

The evidence above explains the high proportion of women delivering in the community from a service supply perspective. But is this out of choice or coercion? Indeed, most of the factors are external and associated with the negative perception of hospitals and clinics. An extensive analysis of government’s policy ambivalence around community TBAs concluded that there is a need to embrace their services [24]. In this study, we hypothesize a theory of the increasing pull of the community maternal care services rather than increasing push of health care facilities. We move from answering questions on *why women deliver in the community* to *why they prefer it*. This study focusses on community deliveries, which encompass deliveries occurring at clients’ homes or other places where community birth attendants (CBAs) practice their trade. These community birth practitioners include traditional birth attendants (TBAs), whose practices are premised on cultural beliefs and spiritual birth attendants (SBAs), whose practices have a religious basis. Both are sensitive to the cultural and religious maternal care needs of the clientele, which modern health care is largely blind to. It should be noted that ‘birth attendant’ is somewhat limited for this study because the practitioners cover antenatal, delivery and postnatal care as opposed to birth alone. We therefore use ‘community maternal care provider’ as a better and all-encompassing term.

## Methods

### Study location

This study is based on findings from 5 districts of Zimbabwe that were selected on the basis of reported prevalence of apostolic sects, scarcity of health facilities and long distances to health facilities. The researchers also knew that these districts had huge rural populations that had been resettled in areas without clinics during the fast-track land reform programme. These five districts are among many more in a broader study on the determinants of maternal mortality in Zimbabwe. They are Nyanga, Makonde, Insiza, Marondera and Mt. Darwin, all representing 5 rural provinces out of 8 in Zimbabwe. In these locations data were collected through focus group discussions and key informant interviews as described below.

### Focus group discussions (FGDs)

We conducted 12 focus group discussions (FGDs) each with between eight and twelve child-bearing women aged 14 to 49 years, who had recently delivered or had a child under two years of age. Women selected for participation were those who had delivered outside a health facility, either at home or a CBA’s place. FGDs were conducted at rural health centres with women who had come for postnatal care services. The focus group had between eight and twelve members each and the smallest group was at a satellite clinic in a resettlement area in Makonde. At least two FGDs were conducted in each district, except Nyanga and Insiza, which had three groups each. At any given time, the proportion of women in the community who have a child below two years of age is very low. To get a sufficient number of participants at one point and time, the researchers visited, by prior arrangements with clinic staff, facilities that had scheduled days of postnatal care.

Most rural clinics dedicate either Tuesday, Thursday or Friday for maternal and new-born care services. These days are generally referred to as *‘mazuva eskero’* (days on which babies are weighed on the scale), although a broader range of services is offered on these visits. These days are usually selected to coincide with the cultural holiday or Sabbath *(chisi)*–a day of rest in a week that is enforced by village heads. On such days, women rest from their routine household chores and agricultural activities, which affords them time for maternal or baby care activities, including visiting the clinic.

The researchers worked with clinic staff to identify women who had delivered outside a health facility by using the women’s clinic records, baby health cards and verbal confirmation. Those satisfying the inclusion criteria were provided with information on the study and requested to participate in a focus group discussion. Verbal consent was requested before participation. This purposive sampling approach was selected principally for its convenience.

FGDs were selected for this study because they enabled participants to openly discuss their opinions and personal experiences. They were facilitated by the researcher with the help of two research assistants, who were recording and translating the conversations. FGDs enabled the researcher (corresponding author) to quickly identify core issues of the subject, observe the reactions of participants and explore new or expected information. They also allowed participants to respond in their own words. Although, personal biases cannot be completely ruled out on both researcher and participants, to minimize this we ensured that group membership included people of different interests and captured data as objectively as possible. A structured FGD guide was used and it covered pregnancy, delivery and post-delivery practices.

### Key informant interviews (KIIs)

This study also used eight (8) key informant interviews (KIIs) that were conducted with female community maternal care providers. Almost exclusively, attendance at delivery is a cultural preserve of women. KIIs were chosen because of their ability to extract detailed information from people with experiential knowledge of a social phenomenon. As a result, four (4) traditional birth attendants (TBAs) and four (4) spiritual birth attendants (SBAs) were interviewed in order to get the cultural and religious perspectives associated with community deliveries. Using a structured KII guide, the researchers tried to extract more details on the subject of community deliveries than would ordinarily be obtained using other approaches. The opinions of community-based maternal service providers were triangulated with those of the beneficiaries to crystalize the story. In this way, the supply and demand issues were analysed together to give one nuanced story on community deliveries.

### Data management and analysis

In all the four Mashonaland districts, namely Makonde, Marondera, Mt. Darwin and Nyanga, data were collected using the Shona language while both Ndebele and Shona languages were used in Insiza district of Matabeleland North province. Data collection was conducted between 1 February and 19 August, 2016. With the help of assistants, data were captured through extensive note-taking and audio-taping. These data were transcribed, translated and entered into Ethnography software. From the transcriptions, the authors identified the emerging themes linked to maternal health beliefs, attitudes and practices around choice of place of delivery. Through content analysis, the themes were coded manually. In Ethnography, data were sifted through, codes sorted and transcriptions summarised. The output displayed the relationships in the form of a tree diagram, where the trunks represented themes while the branches represented the identified issues around a particular theme. We followed three themes around community, place of delivery and birth attendant.

### Ethical issues

The study was approved by the Medical Research Council of Zimbabwe (Reference Number: MRCZ/B/423). It respected freedom to participate and adhered to research principles pertaining to privacy and confidentiality. Participants in this study provided verbal consent to respond to questions in the focus group discussions and key-informant interviews and to be audio-taped. The study involved two teenage mothers who were younger than 18 years. We sought verbal consent from the mothers/guardians, who had accompanied them to the clinic. It is customary for a young mother to be attended to always by a mother, aunt or mother-in-law. Flexibility was provided for them not to respond to questions that they were not comfortable with. Verbal consent was selected, firstly because the explanation for the purpose of and expectations from the interviews was done verbally. Secondly, some respondents requested that they did not want the interviews to appear very formal and serious, as would happen if consent forms were written and signed. Verbal consent was therefore audio-taped as provided for and approved by the Medical Research Council of Zimbabwe. It was also explained that participation was voluntary and withdrawal could be done at any time with no negative repercussions.

## Results

### Sample characteristics

The methodology resulted in an opportunistic sample that had twelve focus group discussions (FGDs) and eight key informants interviews (KIIs), all from the selected rural areas. The educational levels of FGD participants ranged from primary to secondary. All the eight in-depth interviews were conducted with female community maternity health providers. These providers included four traditional (TBAs) and four religious birth attendants (SBAs) who were identified through the referral technique. Referrals were made by women who were intercepted at health facilities for FGDs, health care workers or other community members. The ages of the key informants ranged from 35 to 74 years, while FGD participants ranged from 14 to 49 years. Table 1 below summarizes the demographic characteristics of the FGD participants and key informants.

**Table 1:** Socio-demographic characteristics of study participants.

	Number	Location	Sex/Type	Reported Ages	Educational Level	Marital Status
a) FGD Participants	12 groups (108 participants)	All rural	All female	Range: 14–49 years	39 completed O-level; 58 completed primary but not O-Level; 11 did not complete primary	86 married/cohabiting; 15 polygamous; 7 not married.
b) Key informants	8	All rural	4 TBAs 4 SBAs	Range: 35–74 years	2 completed O-level; 4 completed primary; 2 did not complete primary (eldest)	2 widowed (eldest); 3 married/co-habiting; 3 not established but not living with husband

The study findings will be presented in the following thematic sections. Relevant citations from the raw data will be used to convey the major thoughts coming from FGDs and KIIs.

### Lower transport costs

Pregnant women tend to consider the reachability of place of delivery when deciding on place of delivery. One key element is the cost of transportation. Results show that delivering in the community is preferred because it is less costly compared to delivering in a health facility. Consider a typical remark below, from an FGD in Nyanga:

*‘*When a woman’s time comes [to deliver], going to the clinic requires cash for transport. Public transport is not easy to use so we get help from local businessmen, teachers and other civil servants who own cars. It is not easy to negotiate for later payment, especially if the car-owner asks for cash to cover fuel costs. In any case, most car owners demand cash because they know the majority will take long to pay or may never pay at all’

(Focus Group Discussion, Nyanga)

Probing around this aspect, it was revealed that while transport fares are standard and prescribed along the country’s major highways, the cost per kilometre rises exponentially in rural dirt roads, which are usually serviced by unlicensed and unregistered taxis. The majority of these taxis are not roadworthy but operate on rural routes to cater for the huge unmet demand for transport. This is because most rural routes do not have a regular bus service or only have it seasonally owing to bad road conditions. Since formal bus service providers avoid such routes, unregistered taxis operators find them easy to penetrate. There is no competition for passengers. Furthermore, the routes are highly profitable because there are no police roadblocks, hence no traffic fines, which tend to increase operational costs and cut on profits. The emergence of unregulated transport services along these routes means the charges are also unregulated and always tend to be higher than normal. It also means the operators can monopolize routes or form cartels, which tend to put the cost of transport way beyond the means of the majority.

Further to this problem and, because the country is officially using a multiple currency system, dominated by the United States dollar, liquidity challenges among the rural poor usher new challenges of their own, for which there are no easy or immediate solutions. For example, the lack of smaller $US-denominations means the minimum charge for any distance travelled is US$1. This leads to transport inflation per unit of distance travelled, unlike in urban areas where liquidity is better and transport fares are better regulated. Ultimately, the rural traveller is left alone to deal with the problems of market failure. During a focus group discussion in Marondera, a participant explained that:

‘Any short distance here costs a minimum of one dollar by kombi [taxi]. The transport operators say they do not have change. So people choose to walk the shorter distances. In emergencies, they can use a wheel-barrow or ox-drawn cart to take the woman to the mission [hospital]. Some hire cars but that means pledging a goat or cow as payment, if they can’t pay in cash. So it’s either you choose to go through all that trouble or you get assistance from *Mbuya Nyamukuta* (TBA) or *Mai Muponesi* from the church (SBA)’

(Focus Group Discussion, Marondera)

The above sentiments convey the challenges associated with transportation to a clinic or hospital during a pregnancy-related emergency. The first barrier, it appears, is high cost of fares. The options of walking, using wheel-barrows or ox-drawn carts indicate a real desire to go to a health facility. The thought process depicted above is one of eliminating options on the basis of costs and effort required. Finally, the participant shows that the easiest option available to many is getting services from community providers. At this stage, it is clear that the choice, though not initially the best, is made mainly due to the prevailing difficult circumstances.

However, the women also pointed out that during pregnancy, particularly late pregnancy, walking is not an option. In a state of advanced pregnancy, walking long distances becomes difficult and the need for locomotive transport becomes higher. Thus, the total travel costs are very prohibitive, leading to community deliveries. Often, with community birth attendants, the distances are walkable or the service provider walks to the client’s home. Either way, community birth attendants present as a cheaper and more flexible alternative.

Besides the anticipated transport cost of getting to a rural health facility, there is also the likelihood of referral to a hospital, which comes with an extra cost of transportation. Thus, cost of transportation to a health facility has the known and the unknown but anticipated component, which often comes with referral. At individual and household level, cost calculations of getting to a health facility make institutional delivery highly prohibitive. A focus group discussant in Mt. Darwin pointed out that:

‘When going to the clinic for delivery, you need some money for transport fare, and a possible emergency referral. If they refer you to the hospital, you need money for transport as well as to pay for services. In most cases, we do not have the money, so we try other means, where chances of success are high. At least, no payments are made at the prayer shrine or the TBA but you still get help’

(Focus Group Discussion, Mt. Darwin)

Risky though it appears, focus group discussants pointed out that when services are received from a community birth attendant, chances of being referred further are minimal. Community maternal care providers are said to be ‘*brave*’ in dealing with complications and also for ‘*understanding*’ the socio-economic challenges faced by their clients. There was consensus during a focus group discussion in Makonde when a participant said:

‘Community birth attendants are brave and try their best to assist even in difficult circumstances. Most of them are experienced and can even deal with serious birth and delivery complications. We find it better to get assistance from an experienced person than dealing with emergencies on our own. Besides, they understand the financial challenges we face when going to the clinic and will not simply refer you to a hospital, without trying their best.’

(Focus Group Discussion, Makonde)

However, it was revealed that when a woman’s condition deteriorates further, transport costs are endured nevertheless. In such situations, cars or scotch- or ox-drawn carts are hired. Participants concurred that in such situations, the costs become highly prohibitive, with payments sometimes having to be made in livestock (cows), grain or labour. Focus group members in Insiza generally concurred with a participant who remarked:

‘If everything is normal, delivering in the community cuts transport costs. However, depending on the severity of the complications, you may end up with no choice but to hire a car, even if it’s expensive. If there is no money in the home at that point, as is likely in many cases, negotiations are made with transport owners for flexible payment terms or surety is made in livestock, usually cattle’

(Focus Group Discussion, Insiza).

The quotation above shows that community deliveries are spurred by poor anticipation of complications. It also shows that transportation costs for obstetric emergencies are so high that, when combined with liquidity challenges, a family may be forced to dispose of productive assets. Such desperate coping responses further impoverish the household and make it more vulnerable to future emergencies or an escalation of the current one. Usually, livestock buttresses rural households’ resilience to socio-economic shocks and is also used for household social security. The disposal of livestock to cover transport and maternal hospitalization costs is often a last resort but it happens when serious delays have already occurred which endanger a woman’s life.

### Lower user fees

With community deliveries, user fees are minimal and in some cases non-existent. Where clients are charged for services, some costs are indiscernible because service providers charge for tangible and visible items and products used in providing the service, unlike health facilities which charge consultation and professional fees. One TBA claimed that she had received special training from the government and practised as a means of earning a living. As such, she charges for her services, depending on the severity of the problem dealt with. She explained that:

‘Some years ago soon after independence we [TBAs] used to work as resident community nurses. We were trained by the government and given practicing certificates. Then later the government told us not to deliver babies at home but I still practice because this is my only way of earning a living. So I charge a small fee for my services. When I deliver a woman who has no complication, I charge a hen (US$7). If she experiences a minor complication, I charge a she-goat (US$30). If she comes very late and the baby is stuck in the birth canal, I charge a cow (US$300) for pulling it out (*kupindira*). Female livestock enable me to grow my own herd.’

(Personal Interview with a TBA, Nyanga)

The perception of ‘lower’ user fees is difficult to ascertain from the above quotation, but it appears that cash charges are perceived as being more expensive than livestock. Nevertheless, decisions are made on such perceptions, though they may not be true. Most importantly, it is highly probable that cost comparisons are done relative to the cost models used by clinics and hospitals. It is also likely that women calculate user fees on a cumulative rather than itemized basis. As such, the cost of services at health facilities tend to be higher as they are construed in the light of transportation and other social or opportunity costs.

The key informant’s sentiments also indicate that she received formal training, which is a basis for charging professional fees. Other traditional birth attendants claimed that their practice is a vocation more than a profession. These insisted that they were called by the ancestors to help people and they never directly charge for their services. However, they do accept tokens of appreciation. As such, a majority of poor rural women who cannot afford prescribed fees tend to use these practitioners. One such community birth attendant in Marondera explained:

‘I am a member of this community and I have this special skill in delivering babies. It’s one of the things I do for fellow villagers and it is very difficult to charge. I also receive help from them in different forms so I charge nothing. But if someone wishes to appreciate my services with a gift that is fine. Most famous people you see around came through these hands of mine *[showing her palms*] and even in their adult life they still come back with various gifts to thank me for leading them into this life’.

(Personal Interview with a TBA, Marondera).

Similarly, spiritual birth attendants also claim to be called by God to assist people, hence do not charge for services. They explained that, when offered to fellow church members, their services are an extension of God’s blessing to His redeemed people. When offered to non-members of the church, it is God’s favour extended to them. Such maternal care services are treated as a form of evangelism since benefactors are encouraged to join the church if satisfied with the results. In Mt. Darwin, a spiritual birth attendant explained that:

‘I do not charge women for the services that I give them because I receive instructions on how to deal with the problems from the Holy Spirit. When I pray for a woman and she sees positive results, how can I charge for prayer? The Lord instructed us to give our services freely because He also gave us the gifts freely. However, they can bring offerings or tithes to thank the Lord. We want them to become members of our church so that we continuously help them with prayers and teachings to avoid similar problems in future.’

(Personal Interview with a SBA, Makonde)

Past users of the community-based delivery services feel there is equity and value for money. Compared to clinics and hospitals, community birth attendants charge no administrative costs which are not directly linked to services offered. At rural clinics, women are charged US$10 at booking, which is supposed to cover antenatal and uncomplicated delivery services. At municipal clinics in urban areas, the booking fee is US$50 for a similar range of services. Any extra costs that come with a complicated delivery are borne by the client, in addition to the booking fee.

The booking fee itself is not properly understood by clients. Results show that some women think booking is simply a notice given to the clinic before going there to deliver. As such, they feel that paying money to get recorded in a clinic register for delivery not only lacks sense but also increases the cost of institutional delivery. A focus group discussant in Nyanga remarked:

‘For us booking means telling the nurses that we may come to the clinic to deliver, so that they record us in their books. Unfortunately, they ask us to pay $10 and they want it in cash. I don’t understand why we are charged to be recorded. It looks like they make their own money from us. Because we don’t have the money, we don’t book and because we don’t book, we don’t even bother to go and deliver at the clinic in case they ask for the money.’

(Focus Group Discussion, Nyanga)

The quotation above shows that women are conscious of the cumulative fees charged for delivery purposes. As such, all perceived and real costs make facility deliveries costly. On the other hand, they believe that community birth attendants offer huge discounts as they do much more on benevolence than business terms. Even when they charge a fee, clients appreciate that they collapse numerous services into one charge. In contrast, health facilities itemise their bills for every cost incurred, thereby making their charges perceptibly high. A focus group discussant in Makonde, with the consensus of the group, observed that:

‘Community birth attendants don’t charge for too many things as clinics do. They can ask you to appreciate anyhow you wish or they charge one flat amount. At the clinic, you pay for the card [*registration*], tablets, medicines and consumables. You pay for every little thing; they just fall short of charging for their water and toilets. At one time, we paid US$10 for the card [registration] only to be told to go and get services from the district hospital’.

(Focus Group Discussion, Makonde).

The sentiment above shows that community service providers appear cheaper due to the way charges are administered. They convey full value for money unlike detailed and itemized bills used by health facilities.

In light of the above, community birth attendants are believed to empathise with clients and therefore charge ‘fairly’, unlike clinics that charge prescribed fees, which are not linked to the services provided. Clients feel community maternal service providers can advance their services and demand payments later whereas health facilities use a prepayment approach. The remarks of a focus group participant in Insiza below relays this feeling.

‘Community birth attendants charge fairly because they help you first and discuss the charges and terms of payment later. At the clinic or hospital, it is quite the opposite–money first before service’.

(Focus Group Discussion, Insiza)

The sentiment above reveals that clients feel that health facilities do not empathize with clients. It shows that health facilities are concerned more with money than clients’ well-being. It also shows that at the health facility payment modalities cannot be negotiated if money is not available. What this means is that when pregnancy complications arise in the community, people are likely to refer them to community maternal care providers than a health facility.

### More flexible payment terms

When community service providers charge for services, their terms are perceived to be very flexible. Firstly, they do not demand to be paid in cash and clients find this to be very accommodative, especially in the context of an illiquid economy, high unemployment and other socio-economic challenges. Secondly, it also means emergencies are attended to first before discussions about payment. Consider the following remark by a group discussant in Mt. Darwin:

‘If you don’t have money to pay, *Mbuya* [TBA] accepts chickens or goats. If she performs some complicated procedure like *kupindira* (pulling the baby out in the event of a prolonged delivery) a cow may be required. She also accepts payment in labour, well after delivery. At times, a husband can later perform menial tasks for her as payment’.

[Focus Group Discussion, Mt. Darwin].

Getting money is reportedly a huge challenge for many people affected by austere macro-economic conditions and payment in kind is a welcome option. The quotation above shows that community service providers accept livestock, poultry, grain, labour and other social favours as payment. The above quotation also expresses satisfaction with the flexibility of payment terms since clients can pay ‘well after delivery’. It also shows that community maternal care providers are more concerned about the client’s welfare more than money or payment. This is greatly desired and appreciated by the clients, unlike health facilities that demand cash at entry.

In times of emergency, this flexibility is seen to be particularly helpful. An emergency is usually a time of panic and vulnerability to manipulation. It is a time when the victim is usually in need of the highest possible level of support from the family, the community and the health delivery system. It is this kind of understanding that women are looking for when obstetric emergencies arise. This was explained below by a participant during a focus group discussion in Makonde:

‘When a woman starts bleeding at home during labour, you cannot afford to waste any more time looking for money. In many cases, it is usually not there anyway because emergencies usually strike when there is no money at hand. In such cases, one should be able to get help simply on the promise of later payment but most people prefer showing something tangible, like pledging livestock or grain.’

(Focus Group Discussion, Makonde)

Besides the flexibility in currency of payment, community service providers are also flexible in repayment time-frames. They accept long-term debt-settlement plans and instalments. Poor people in rural areas sometimes do not have problems with the amount charged for services. However, they tend to struggle with the requirements to pay in cash or other sophisticated cash transfer modalities. Secondly, they also struggle with requirements to make single settlements, especially when they do not have the capacity to generate the money at one time. The findings indicate a desire for more time to pay in small instalments until the whole amount is cleared, which is possible with community birth attendants but not with health facilities.

Another desirable aspect of negotiated payment terms is that they accommodate payment-by-results, whereby clients pay service providers after seeing and confirming the desired results. This helps to satisfy the desire for value for money by clients, which is not possible with health facilities. Thus, community service providers can be paid later after the client confirms the desired results. A traditional birth attendant in Marondera indicated that they also like this approach and explained that:

‘When a client is brought here for services, we don’t talk about money or payments first. Most of them are friends, relatives or people that we know so they can make a pledge to pay later and we accept that and provide services. For relatives, it’s difficult to charge so I ask them to pay when they are happy with my services, after survival.’

(Personal Interview with a TBA, Marondera).

The sentiments above show that community maternal care providers are results-oriented and not motivated by gain. As such, they win the trust and confidence of clients because they provide services not as a business but through a desire to see their clients happy.

The method of payment by results is the most preferred, unlike in clinics and hospitals where clients pay administration and consultation fees before receiving services. Besides helping the client alone, negotiated payment terms also benefit service providers, hence strengthen the localized economy. For example, to some community maternal care providers, grain payments are the preferred medium of exchange. Given that most of their time is spent in maternal care, they rarely find time for agricultural engagements. So the flexibility of payment terms benefits both service providers and their clients.

### Lower social and opportunity costs

Apart from the numerous financial costs avoided by delivering in the community, opportunity costs are significantly reduced. Results show that going to a health facility means foregoing numerous household chores and responsibilities, which are crucial to the welfare of the family. Worse still, admission in a clinic, hospital or a maternity waiting home denies both the family and the woman the emotional and psychosocial benefits of being together as a family. Usually, the other young children are left in the custody of a relative or maid at a huge social cost as both “miss” each other’s warmth and company. As the children miss the warmth of a mother’s presence, the mother also gets worried about the welfare of her children. Thus, delivering in a health facility means that a woman is not with her family for several days or weeks. Young children can go through an experience similar to maternal orphan-hood while the husband also goes through a period similar to widow-hood. The only difference is that in this case there is the hope of a mother’s or wife’s return. Thus, the social and emotional effects of a woman’s temporary withdrawal from her family are as broad as they are diverse. Consider this remark by a spiritual birth attendant in Makonde:

‘Taking away a mother from the home means doom for the whole household. The young children suffer, the husband suffers and the woman herself also suffers. This is why we say ‘*musha mukadzi*’ [a woman makes a home].’

(Personal Interview with a SBA, Makonde)

The key informant’s sentiments above highlight some of the social problems associated with delivering away from the community. It is clear that all members of the family are deprived somehow, hence affecting the whole household. The salient statement is that, as much as possible, a woman should be close to her family. In addition, when a woman is admitted for maternity care away from her home, she is basically creating another household structure, with similar basic requirements as the principal household. With a strong consensus from colleagues, a group discussant in Mt. Darwin observed that:

‘There are common resources like bath soap and food that are shared by family members. If a pregnant woman is hospitalized or is at a waiting home, that creates another household. If the husband is working away from home, it means the breadwinner has to sustain three households at the same time. Cooking in one pot saves family resources’

(Focus Group Discussion, Mt. Darwin).

This quotation reveals that the hospitalization of a woman for delivery purposes can temporarily dualise or triplicate the household structure, against static resources. Apart from straining the household economy, it can also stretch a woman’s emotional capacity as a pillar of the household. An alternative is to leave the younger children with a relative or friend but this also strains the receiving household. As much as possible, women avoid burdening others with their child-care responsibilities and, on that basis, may forego institutional delivery.

Women also expressed concern about leaving their husbands alone for prolonged periods of time, arguing that it increases the likelihood of unfaithfulness, multiple sexual partnerships, HIV infection, family malfunction and divorce. Most women feel that it is them who make their husbands a little responsible. They argue that, on their own, men cannot effectively run a household or manage a family. Thus, men cannot tolerate a temporary gap left by a woman as they want to fill it quickly by courting other women. Consider this comment, which was lively supported during a focus group discussion in Insiza:

‘Men are like children. They need to be watched every time. If you leave them alone, they will be taken by other women’.

(Focus Group Discussion, Insiza).

Given a choice, women would prioritize their marriages over delivering in a health facility to avoid marital discord. Culturally and religiously, it is believed that a wise woman makes sure that her marriage remains intact. To abandon the home and risk a marriage on account of a pregnancy-related admission at the hospital looks less prudent. This phenomenon is related to fears of losing family connection presented below.

### Desire for home-based services

Community maternal care providers can allow clients to go back and check on the welfare of their families. Alternatively, the service provider can stay at the client’s home while offering services (home-based services). Such maternal care services are greatly desired as they reduce the financial and social costs highlighted above. A spiritual birth attendant in Makonde explained that:

‘There are cases when a pregnant woman has spiritual issues that need to be dealt with at base. I can move into her home, stay there for a few days to provide her with care while dealing with the spiritual issues in her household.’

(Personal Interview with a SBA, Makonde).

The above quote shows that community service providers are willing to provide mobile maternity or new-born care at the client’s premises. However, if there are no signs of imminent danger, the service provider commutes from her home. This is a preferred arrangement particularly when the woman has other small children who need her presence and care. Participants concurred that this arrangement, where they have complete control over their households, brought them peace of mind during pregnancy and delivery.

Women hinted that they would be particularly concerned about the welfare of their young children if they were away from home for prolonged periods of time, even with regular mobile phone communication. Indeed, most pointed out that even the most trusted relatives would not be able to take care of their households as they would do themselves. These fears are exacerbated because the day of delivery is not known with certainty so several days can be spent awaiting delivery at the health facility.

Compared with health facilities where freedom of movement is relinquished upon admission, the community maternal care providers allow for contact with the family. Delivering in the community has no restrictions or social curfews. This was revealed by an apostolic sect member during a focus group discussion in Insiza:

‘Community birth attendants know that we have families and allow us to see them regularly. When we go to the birth camp (*chitsidzo*), the church allows us to take our young children with us. We can also visit them when we feel like or they can visit us. The hospital is like a prison because they won’t let you go out to see your children until after discharge. You will miss your children and you will also be home-sick. You can’t go and visit friends, relatives or church mates. You can’t even attend funerals or weddings either’

(Focus Group Discussion, Insiza).

The quotation above shows that community service providers strive to maintain the social life of their clients. They also make provisions for continuous contact with the family. The statement also shows that a client’s desire to attend and participate in social functions is respected and facilitated. With hospitalization, clients can no longer practice their religious or cultural activities, unlike in the community. Further, visits from or to friends and relatives are screened, regulated and sanctioned by the hospital system, yet in the community there are no social quarantines. Thus, hospitalization tends to place a woman in the sick role, which tends to limit or temporarily withdraw her social rights.

### Religiously and culturally sensitive services

The study also revealed that pregnant women and young children must be given culturally- and religiously-sensitive services, unlike those given in health facilities. Although not exactly in the sick-role, a pregnant woman is regarded as a special type of patient who deserves special care. The concept of maternal care in Zimbabwe is generally shrouded in spiritual sensitivity. It is generally believed that the unborn child is on the way from the spiritual realm and maternal care must acknowledge that. As such, there is etiquette and custom around maternal care that is usually disregarded and violated during institutional delivery. A traditional birth attendant in Makonde explained that:

‘Once a woman gets pregnant, she is no longer herself. Even her body changes and adapts to the new role of making another life. There are a number of issues that must be considered in her diet, clothing, habits, conduct and much more. Remember that everything she does is for herself and the baby inside her, over which the ancestors are watching. There are certain customs that must be observed to help the baby on its way from the world of the spirits (*nyikadzimu*)’

(Personal Interview with a TBA, Makonde)

The statement above reveals the strong spirituality typical of Zimbabwean people on matters of birth. For the majority, life is more than physical. Therefore, the spiritual aspects of life must never be ignored or totally replaced with modern health science. This belief is so strong that even the women who once delivered in a health facility reported practising these customs during and after hospitalization.

The spirituality associated with pregnancy and child-birth is carried forward into the post-delivery period as similar customs are observed for the new born. It is believed that doing otherwise may lead to misfortune to either the mother or the baby. A spiritual birth attendant in Nyanga explained that:

‘A pregnant woman is a special person because she is a meeting place of the gods and humanity. Inside her, the gods give the world another life, another person… She needs special care as much as her baby.’

(Personal Interview with a SBA, Nyanga)

The statement above reveals that spirituality surrounds the perinatal period, which can affect both the mother and the baby. The community is the best place for observance of cultural and religious beliefs than in a health facility. Some women, even among those with secondary education, indicated that they would rather observe cultural or religious taboos than suffer a misfortune or invite it on their baby. Although desire for a healthy baby is one of the motivations for seeking postnatal care at health facilities, they do not trust the health care system with the unseen baby. This is why the rely more on the spiritual.

The need to observe cultural and religious practices also puts pressure on women to deliver in the community because there is freedom to do so. As a response to the spiritual demands, prayers and rituals are performed on the pregnant woman, as explained below by a spiritual birth attendant in Makonde:

‘A baby does not come from the womb as you would naturally think, but from the spiritual realm. To receive the baby in a healthy state, the woman must be assisted spiritually to receive her gift. This involves routine prayer, fasting and anointing by spiritual leaders.’

(Personal Interview with a SBA, Makonde).

Clients who believe in such an explanation for the genesis of life are less likely to deliver in a health facility, where routine ‘prayers, fasting and anointing by spiritual leaders’ would not be possible.

### Motivation of past experiences

Results show that previous successful community deliveries tend to motivate women to repeated use of community maternal care services. Such positive previous outcomes influence future decisions on place of delivery for clients. In Insiza, a focus group discussant said:

‘I delivered my first boy at the mission (hospital) because the nurse at the local clinic said first babies must be delivered at the hospital. After all the trouble of going there, I experienced no problems at all. After delivering early in the morning, by evening I was at home. So, for my second boy I didn’t even bother. I just went to *Mbuya* (TBA) for help and she was very good. For this one (pointing at 6-week old baby) I also went to *Mbuya* (TBA).’

(Focus Group Discussion, Insiza)

It is apparent that the only institutional delivery that the respondent experienced was because the nurse recommended it during antenatal care. However, while exercising personal choice, the client opted for community delivery for her second and third deliveries. It is most likely that, like many others, she can go back to a traditional birth attendant for her next delivery. It is pertinent to note that she highlighted ‘trouble’ in getting to the health facility for her first delivery.

Previous success in community deliveries also tends to build the confidence of traditional and spiritual birth attendants to promote and market their services. However, what emerged during the interviews is that much of their success in birth attendance is guaranteed by careful selection of clients. A traditional birth attendant in Marondera explained that:

‘If a woman comes here for help on time, she gets adequate help. I have never lost a client (to death) here. However, there are some who present late with serious problems. These I immediately refer to the clinic. I don’t want deaths here because it spoils my name and can cause bad luck (*munyama*). It can also result in *ngozi* (vengeful spirits) as the dead woman’s spirit will accuse me of not helping her enough.’

(Personal interview with traditional birth attendant, Marondera).

The success of community maternal care providers, although premised on careful client selection, gives a false promise of safety to clients. There is likely to be personal referral of new clients by previous users of the services, which may have an element of service marketing, whereby the referrer focusses more on the positive aspects of the service than the negative. In cases where users and providers of community delivery services have positive past experiences, it is difficult to convince them to switch to institutional care.

### Conceptualization of disease and health

Another reason why women prefer community deliveries is their understanding of the origin of maternal complications and the concept of hospital care. First, there is a belief that when a pregnancy is planned for and the baby is desired by the woman, there should be no misfortune during delivery. As such, complications are neither suspected nor expected, hence a woman can successfully delivery anywhere with minimum assistance. Consider this remark from a focus group discussion in Nyanga:

‘Look at animals–they don’t need hospitals to deliver their young. Birth is God’s law and when it comes with problems, it is cursed. What you need is exorcism by a traditional healer or a prophet (spiritual healer). This is why you see that even with the best doctors, one can still die. So you better work with someone who can plead with God and the ancestors for safe delivery.’

(Focus Group Discussion, Nyanga)

The quotation above shows that the pathology of disease and delivery complications is conceived to have a spiritual basis rather than a biological one. With such beliefs women prefer community maternal care providers who can deal with the imminent spiritual problems around the delivery period. The analogy of nature and wild animals also shows that the social construction of disease and health borrows more from nature. Also, interpretations of maternal complications and possible responses may also follow suit.

Secondly, another emerging issue is the perception of health facilities as treatment centres rather than places for specialized management of pregnancy, labour and delivery. Pregnancy itself is not conceived as a disease and therefore rural women feel that there is no need to go to a health facility. The concept of preventive hospitalization is more difficult to grasp than curative hospitalization. During a focus group discussion in Makonde, there was general consensus when a participant remarked:

‘Nurses tell us to come here (to the clinic) when pregnant because they want to check our health and that of the baby. They don’t know that we rarely have time for that and we know they can’t see what is inside because they are not God. The clinic is for treating diseases. Coming here just for delivery does not make sense because we can deliver at home. Besides, when you come for delivery only the nurses will harass you saying ‘where were you all this time’?’

(Focus Group Discussion, Makonde)

From the above, it can be seen that because pregnancy is not construed as a disease, hospitalization is deemed unnecessary because no treatment is done. Institutional delivery is seen as a desire for management of the birth, which is assumed to be possible in the community. It is also evident that presenting at health facilities only during labour and delivery periods is frowned upon by health care workers, who expect women to start with antenatal care.

## Discussion

The results of this qualitative investigation show that although Zimbabwean policy discourages community deliveries, they are on the rise. Secondly, although community birth attendants are outlawed, they are still in practice. Given a choice, poor women in marginalized rural communities would readily choose community maternity service providers over health facilities. The public health care system is structured, set and rigid to an extent where it fails to adjust to the needs of clients. Yet the circumstances that pregnant women find themselves present unpredictable challenges that require flexibility by service providers.

The ZDHS showed that lack of money to pay for treatment was cited as a barrier to health care utilization by 50% of respondents [5]. However, these surveys do not examine how lack of money translates into a decision to deliver in the community. It is expected that lack of money, coupled with a huge motivation for institutional delivery, results in women presenting at health facilities either to be turned away or to later accumulate unpaid bills. This is not the case and this study reveals that disadvantaged communities tend to magnify maternity costs. They are aware of, closer and more sensitive to the other not-so-obvious costs, which make hospitalization costs perceptibly unmanageable. Thus, hospitalization costs are viewed in light of general financial challenges at the household level. It can be seen that abolishment of user fees alone will not translate into increased health care utilization.

The ZDHS further shows that financial problems were cited by 68% of women who had five or more living children who failed to utilize health facilities [5]. This study revealed that a huge family reduces the amount of household resources that can be availed for maternal care. Further, it illuminates the fact that most rural households have no resources reserved for maternal emergencies. Thus, as the choice for institutional delivery increases demands against static household resources or non-existent reserves, community delivery becomes a better option. It also emerged that, for most marginalized families, financial barriers are perceived rather than confirmed. The knowledge that health facilities charge fees and require payments to be done before services can be offered tends to delay the decision to seek help. This leads people to seek alternative medicine, including traditional and religious options.

Apart from financial costs, awareness of the opportunity costs of hospitalization also influences decision making. While financial problems are an obvious barrier, this study revealed that married women with younger children found it difficult to manage their households if hospitalized for delivery. First, they are concerned about the welfare of their young children in their absence. Secondly, they are concerned about infidelity by the husbands. Lastly, if they try to cut the duration of hospitalization by reporting late, challenges associated with delays in seeking hospital care begin to set in. As a way of managing the crisis and balancing the priorities, they find it easier to deliver in the community, where such fears are allayed.

This study showed that the main barriers to institutional deliveries revolve around poverty, which is common among marginalized rural populations. Even rural households that are not impoverished find themselves in a taxing environment where the unit cost of services and products are much higher than in urban areas. This is consistent with ZDHS findings, which showed that cost-related barriers were prevalent among respondents who were divorced, separated or widowed (64%), resided in rural areas (59%), lived in Matabeleland South (65%), had no education (75%) or were in the lowest wealth quintile [5]. Most of these social deprivations are prevalent in rural areas and for some individuals and households, there are multiple and overlapping deprivations.

Related to costs, financial problems and poverty discussed above, community deliveries have the appeal of flexible payment terms since clients can pay for services in a ‘currency’ favourable to them, which can include grain, livestock, poultry and manual labour. These forms of payment, although preferred, cannot be used in the public health system. The formal health care system is designed on a prepayment model where payments, in whole or part, have to be made before services are provided. This model is unfavourable during emergencies where there are no pooled resources to draw from. Thus, the rigidities of currency and payment terms in the public and private health system are discriminatory. They tend to differentiate access to maternal health care, but always place the poor at a disadvantage.

It also emerged from this study that poor people in rural areas are not exactly looking for free maternal health care. They are ready, willing and able to pay for health services as demonstrated by their ability to pay community maternal care providers. Although the size of payment matters, it seems that community service providers have more favourable terms of payment than health facilities. The administration of payments by community maternal care providers is perceived to be fair, friendly and favourable. As such, even without complex calculations, clients perceive value for money. This demonstrates that there is an opportunity to make those who can pay for health care pay what they can, on terms favourable to them.

The requirement by health facilities for cash and dollar-denominated payments is a barrier. To deliver in an institution, clients have to first convert their wealth or possessions into cash. In doing so they bear huge transactional costs which may include unfair prices or exchange rates, particularly in emergencies where buyers can bargain from a position of strength. Thus, the seller, who in most cases owns the emergency’ loses value. Further, converting goods, labour or livestock into cash as required by the health system involves loss of time, which makes it least preferred in emergencies. Community service providers are more flexible, hence more appealing in times of emergencies.

More evidence from the ZDHS shows that distance to health facility was cited more by rural (49%) than urban women (11%) [5]. This study demonstrates that women in rural areas face multiple challenges that can make community deliveries preferable. While this is related to transport costs as discussed earlier, another dimension is that distance is related to social detachment between a woman and her family. Culturally, mothers are regarded as the glue that keeps the families together. A common Shona idiom is *‘musha mukadzi’*, which means the woman makes the home for both the children and the man. In late pregnancy, the costs of transportation increases significantly since, for long distances, private transport has to be hired. Community deliveries therefore address problems related to distance to health facilities, financial costs and emotional detachment.

It was pointed out that although Zimbabwe’s MMR recently started to decline, community deliveries remained on the ascent [8,9,11]. This pattern corroborates earlier studies that showed that community deliveries are not necessarily associated with higher maternal mortality rates [10]. This study involved women who had delivered in the community but survived maternal mortality. Community delivery was a choice for them, although it may have been influenced by adverse circumstances. It is most likely that, in the absence of significant changes in the formal health system, the same women are candidates for the next community delivery. Rosmans et al. [11] proposed that this can be partly due to self-screening, whereby women feel competent to deliver without skilled attendance at the health facility. This study buttresses these assertions in that the service beneficiaries report getting better value for money and a huge return on investment than when hospitalized. To encourage women to deliver in health facilities, more must be done to reduce the charm of community service providers. There is a lot that the formal health system in Zimbabwe can learn from the community service providers especially around flexibilities that reduce perceived financial and social costs. Without these adjustments, community deliveries will be a reality for much longer, in spite of the knowledge that they predispose women to maternal death when complications arise.

It is also clear from these findings that the concept of motherhood in a Zimbabwean context subsists in two roles, namely child-bearing and household maintenance. This means married women feel complete and satisfied when able to reproduce and raise children in a stable family unit. Dissonance and role conflict arise when the demands of one responsibility–pregnancy and childbirth—upsets this emotional equilibrium. To restore this balance, women try to minimize the time they are away from home, either by opting for community services or mobile service providers, which removes the need to leave home. Further, the social construction of health facility as a curative treatment centre rather a preventive treatment centre causes other women to avoid using them for pregnancy or delivery, which are not perceived as a sickness or disease.

In light of the findings of this study and the context of the literature review, we make the following recommendations. Firstly, in the long-term, an equitable an inclusive health care system should be developed. It should not exclude poor people in marginalized communities on the basis of distance or user fees. Secondly, noting that community maternal care providers are existent, entrenched, recognized and often preferred, health care policy should strategically partner with them rather than antagonize. For example, they can be engaged to provide basic obstetric care services and refer clients to health facilities. They already have a presence, the trust and acceptance of community members, they speak their language and identify with their circumstances. They would be referral figures for behavioural and social change on making a choice for place of delivery. If not embraced and incentivized for partnership, they can continue to be a passive impediment to institutional deliveries, by offering alternative care parallel to the public health system. Thirdly, the formal health system should become more adaptive, sensitive and responsive to the social, religious and economic needs of the people it serves. Already, insights and lessons can be drawn from what the community maternal care providers are currently doing right. Lastly, this study reveals that poor people are willing and able to pay for health care. Government should devise ways of harnessing this potential into actual maternal health insurance schemes that are managed by and beneficial to communities. An equity focus in maternal health care programming can help reduce maternal mortality in Zimbabwe.

We acknowledge that the results of this study may be compromised by recruiting women who had delivered in the community from health facilities. This might have introduced selection bias and, in addition, some of the selected women might have coincidentally rather than deliberately experienced community deliveries. Indeed, the study revealed key insights on why women opt for community deliveries, but specific groups, selected by specific socio-demographic characteristics, could possibly show different patterns. Again, our focus on marginalized rural areas only provides the perspectives of rural women who have no other easily accessible and affordable alternatives to maternal health care. As such, we acknowledge that the study misses a crucial component of why women prefer community deliveries. More so because community deliveries also happen in urban areas, farming and mining communities, where health facilities are available and easily accessible.

## Conclusions

The push factors of the health system that act as barriers to health care utilization are largely known. In Zimbabwe they include long distances, perceived high cost of services, lack of equipment, drugs and sundries, poor staffing and bad worker attitudes. However, what is not known is that women are no longer being pushed by the negatives of the health system. Rather, they are responding more to the allure of the community maternal care providers. These are more appealing due to perceptions of low cost of transport and services, flexibility of payment terms, mobility of service providers, promotion of family cohesion and sensitivity to cultural and religious preferences during pregnancy and childbirth. To stem the trend of women choosing to deliver in the community, inequities to health access arising from distance and cost issues have to be addressed. The push factors of the health delivery system also have to be attended to. There is a lot to learn from community birth attendants that can be packaged into modern interventions disbursed through health facilities.
